# Combing the hairball with BioFabric: a new approach for visualization of large networks

**DOI:** 10.1186/1471-2105-13-275

**Published:** 2012-10-27

**Authors:** William JR Longabaugh

**Affiliations:** 1 Institute for Systems Biology, 401 Terry Avenue North, Seattle, WA, 98109-5234, USA

**Keywords:** Visualization, Networks, Open-source, Graph layout

## Abstract

**Background:**

The analysis of large, complex networks is an important aspect of ongoing biological research. Yet there is a need for entirely new, scalable approaches for network visualization that can provide more insight into the structure and function of these complex networks.

**Results:**

To address this need, we have developed a software tool named *BioFabric*, which uses a novel network visualization technique that depicts nodes as one-dimensional *horizontal lines* arranged in unique rows. This is in distinct contrast to the traditional approach that represents nodes as discrete symbols that behave essentially as zero-dimensional points. BioFabric then depicts each edge in the network using a *vertical line* assigned to its own unique column, which spans between the source and target rows, i.e. nodes. This method of displaying the network allows a full-scale view to be organized in a rational fashion; interesting network structures, such as sets of nodes with similar connectivity, can be quickly scanned and visually identified in the full network view, even in networks with well over 100,000 edges. This approach means that the network is being represented as a fundamentally linear, sequential entity, where the horizontal scroll bar provides the basic navigation tool for browsing the entire network.

**Conclusions:**

BioFabric provides a novel and powerful way of looking at any size of network, including very large networks, using horizontal lines to represent nodes and vertical lines to represent edges. It is freely available as an open-source Java application.

## Background

### Traditional network visualization

Despite the increasing importance of analyzing and understanding very large networks of data, the traditional way of visualizing networks has difficulties scaling up, and typically ends up depicting these large networks as “hairballs”. This traditional approach does indeed have a deeply intuitive foundation: nodes are depicted with a shape such as a circle or square, which are then connected by lines or curves that represent the edges. However, although there are many different ways to apply this basic underlying idea [1], it needs to be revisited in light of current and emerging needs for understanding increasingly complex networks.

The traditional way depicting networks has the following characteristics:

•Though nodes are typically *depicted* as small two-dimensional glyphs, they are, in essence, zero-dimensional points positioned in two-dimensional space.

•Edges are shown as lines or curves, i.e. essentially one-dimensional objects, positioned in the same shared two-dimensional space.

•When there are many edges to or from a node, they are all converging on a single zero-dimensional point. Furthermore, since node locations are not constrained, overlapping zones of edge convergence result in unavoidable ambiguity, as do edges that may intersect intervening nodes between the two true endpoints.

•Since edges are arbitrarily positioned, they can easily overlap each other, and invariably create a huge number of arbitrary, meaningless intersections that can completely obscure the paths of individual links.

•The addition of each new edge to the network degrades the existing presentation, as the edge will typically overlap existing network features. This property means the traditional approach is inherently unscalable.

### BioFabric visualization technique

**Figure 1:**
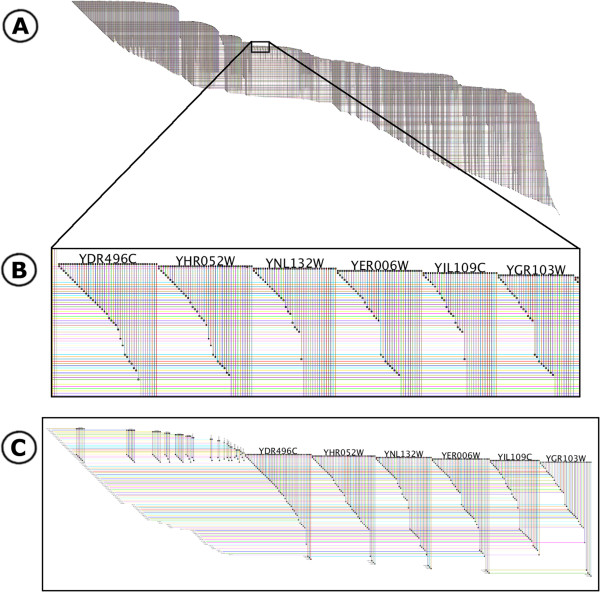
**BioFabric network display.** This is a depiction of the yeastHighQuality.sif data set [3-5] containing over 3000 nodes and 6,800 edges. The key feature of the BioFabric presentation is that nodes are depicted as *horizontal lines*, one per row; edges are presented as vertical lines, each arranged in a unique column. Note how the use of darker colors for rendering edges and lighter colors for rendering nodes insures that the former stand out despite the crossover. **A**) The view of the full network, laid out with the default algorithm. **B**) Detail of network shown boxed in network A, which highlights one advantage of the BioFabric presentation technique: similarities, and differences, in the connectivity of different nodes are immediately apparent. **C**) The six nodes and first neighbors depicted in a *subset view*, where all extra space has been squeezed out, creating a compact presentation that still retains all the relative positioning from the full view. Note how the full inventory of edges incident on the six nodes also includes those on the left originating from higher node rows.

BioFabric tackles the problem of depicting large networks by changing the underlying representation. Figure 1 illustrates how BioFabric renders a network of yeast protein-protein and protein-DNA interactions with over 3,000 nodes and 6,800 links. This is the yeastHighQuality.sif sample network distributed with the Cytoscape [2] download [3]; it is based upon [4] and [5].

The BioFabric approach has the following characteristics:

•The key feature is that nodes are represented as one-dimensional *horizontal* line segments, one per row.

•Edges are represented as one-dimensional *vertical* line segments, one per column, terminating at the two rows associated with the endpoint nodes.

•Both ends of a link are represented as a tiny square. This provides sufficient contrast to make the ends of the link stand out even at large scales. In the case of directed edges, the appropriate end is tagged with an arrowhead.

•Edges are unambiguously represented and *never* overlap. In networks that have multiple edges between the same nodes, i.e. representing different types of relationships, all edges show up clearly.

•As nodes are represented as horizontal lines, there is no requirement that all edges converge upon a single point, allowing for complete flexibility in where a link is drawn. Links can originate, and terminate, anywhere along the length of the node segment. This flexibility introduces the powerful ability to create sets of links that share some semantic property and depict them as discrete groups arranged horizontally in the visualization.

•The addition of a new edge just increases the *width* of the visualization, and does not degrade the existing presentation in any fashion. And increased width can be thought of as simply adding pages to a book; the network is being represented as a fundamentally linear, sequential entity, where the horizontal scroll bar provides the basic navigation tool for addressing the entire network.

•Edges are drawn darker than nodes; this has the effect of emphasizing the links and making them appear to float in front of the nodes. So despite the existence of a vast number of orthogonal intersections, links and nodes are unambiguous.

•The visualization technique produces a distinct *edge wedge* for each node, created by the close-set juxtaposition of the parallel links. The wedge provides clear visual cues about how the node is connected, and how it compares to other similar nodes.

•A set of 32 colors is used, not randomly, but in a repeating cycle to render node and edge segments. Colors are not used to apply semantic meaning to network elements, but are crucial for providing a framework that allows the user to visually trace features over long distances. Also, the use of cycling insures that antialiased rendering will produce larger-scale color patterns that provide useful visual cues even when individual links cannot be discerned.

•Note that the traditional technique overloads the two-dimensional plane, using the *same* space to represent *both* nodes and edges. BioFabric effectively segregates the plane into two separate one-dimensional spaces, and assigns each space to either nodes or edges; the imposition of orthogonality and the use of judicious rendering allow the user to visually distinguish the two. Thus, BioFabric can provide additional clarity of the network structure while using the same underlying two-dimensional resource.

### Previous work

Using lines to depict nodes has appeared previously in the literature. McAllister [6] used the technique to illustrate an algorithm for the *linear arrangement problem* (LAP), which finds an ordering of nodes arranged along a line that minimizes the sum of the edge lengths in the graph. In this instance, it is a natural representation that allows the edges to be clearly shown despite the one-dimensional nature of the problem. Another common use where nodes have a linear representation is in Unified Modeling Language (UML) sequence diagrams, where objects have an associated vertical *lifeline*[7]. However, in that context, the lines are specifically being used to represent the objects over the passage of time.

### Contrast to adjacency matrix

It is also useful to contrast BioFabric with another common method of visually representing a network: an adjacency matrix. For a network of *n* nodes, the matrix is laid out as an *n* x *n* grid of points, symbols, or cells. In general, each node *m* is assigned to both row *m* and column *m*. Each edge in the network between node *r* and node *c* is then depicted with a symbol in row *r* and column *c*. Though this approach has the powerful advantage of being unambiguous, it still suffers from some critical shortcomings:

•The area of the representation increases as *n*^2^.

•Many large networks are sparse; a network with 10^4^ nodes has over 10^8^ possible edges, and thus 10^5^ edges would only have one edge cell filled for every thousand available spots. The depiction of the network is mostly empty space.

•The representation of edges as essentially zero-dimensional points gives them much less visual impact than one-dimensional lines, yet the edges in a network are arguably the essential aspect that needs to be conveyed to the viewer.

### Contrast to power graph analysis

Various other techniques have been employed to try and handle the scalability problem; one such technique is Power Graph Analysis [8]. The method explicitly identifies recurring network motifs (e.g. cliques) and uses simplified graphical representations for these structures that implicitly represent a large number of edges without needing to render them. This clever method can result in a significant edge reduction, but still has the same limitations as the traditional method for the remaining edges that still need to be drawn. Note that BioFabric can use some of these same simplifications, such as symbolic representations of cliques, as well. One planned future enhancement for the software will allow cliques to be represented compactly as multiple endpoints glyphs on a single vertical segment. Variations on this technique could also be used to depict hyperedges.

## Implementation

### Platform and libraries

BioFabric was quickly built using the pre-existing Java code base that has been developed for BioTapestry [9,10], a Java application for modelling and visualizing genetic regulatory networks. Thus, it uses many of the same core Java libraries that BioTapestry is built upon: Java Swing, Java2D, and Java ImageIO.

The Java2D library proved to be an excellent platform for BioFabric development, particularly due to its antialiasing support. This is important because the BioFabric approach is prone to aliasing artifacts: it involves rendering many very tightly spaced parallel lines, which are being drawn with a repeated cycle of colors. In fact, with large networks and full-network zoom levels, there are multiple lines (e.g. tens, hundreds, or more) being rendered through *each pixel*. Yet it was not necessary to spend any development time working on specialized low-level, resolution-dependent pixel coloring code to handle this; the standard Java2D draw() method was sufficient, in combination with setting the corresponding Java2D RenderingHint to VALUE_ANTIALIAS_ON. The only caveat that has cropped up so far is a requirement to use Java 1.6 on Apple Macs to get the desired network display. With Java 1.5 on the Mac, the BioFabric networks appear too light compared to all other platforms (e.g. Windows and Linux), yet this problem disappears using Java 1.6.

### Rendering cache

BioFabric is intended to provide useful visualization of a network with 10^5^ or even 10^6^ edges. In order to keep rendering times down for the large-scale zoom levels, BioFabric starts rendering the network to image buffers in memory as soon as the network is loaded from a file. With the exception of the single top-level zoom image, a grid of image tiles is used to render each zoom value above the level where the program can get adequate performance using immediate mode rendering. After the first two zoom levels are cached, the file load is completed and control passes to the user. From then on, subsequent user pans and zooms are handled using tiles from the image cache. If a needed tile has not yet been generated, a low-resolution tile is created immediately from an available large-scale existing image tile, while the needed final high-resolution tile is queued up for creation on a background thread. Those results are then swapped in as they become available. This approach allows the program to remains responsive even when dealing with large numbers of links and edges, yet the user experience is familiar to users of online resources such as Google Maps [11].

### “Shadow Links” can improve the user’s understanding of the network

BioFabric has two different modes for rendering network edges. In the *standard* mode, each edge appears only once in the network. This has the advantage of being clean, compact, as well as being consistent with the traditional way that networks are depicted: one line is drawn per edge. However, the addition of a *shadow link* mode provides a powerful alternative visualization technique.

**Figure 2:**
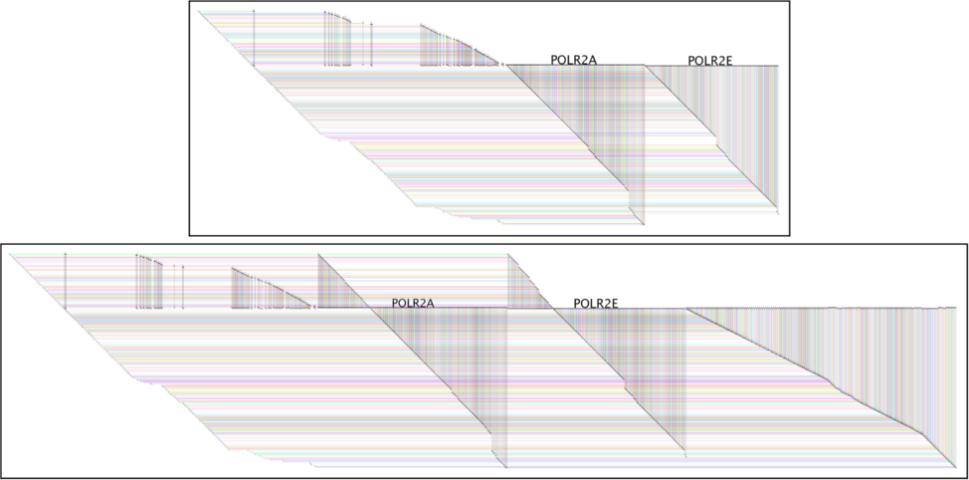
**Shadow links.** Shadow links provide compact, complete views for all of the edges incident on a node. These two subset views are taken from the Human_Interactome_May.sif network [15], and show the same first neighbors of POLR2A and POLR2E. The top image shows the *standard* mode view; every edge appears once. Note that although a prominent node label appears over the large contiguous set of edges associated with the right terminus of each node (to enhance readability), those are *not* all the edges incident on the horizontal node row line. There are also edges on the left that are more closely associated with the distal nodes for those edges. In contrast, the bottom image shows the *shadow link* mode; each edge is has a duplicate shadow link, so that each node in the edge pair can display a copy of the edge as part of a single, labeled *node zone*. This provides a complete inventory of the edges in one location, allowing direct visual comparison of the connectivity of multiple nodes. This particular view of all the edges is provided here just to clarify how the shadow links are situated in the network; note that the bundle of edges on the right side of the lower view are the shadow links connecting to nodes laid out in the rows below POLR2A and POLR2E. The actual default display policy for creating subset views when shadow links are active is to discard the leftmost (true) and rightmost (shadow) links.

Figure 2 illustrates the difference. Given the approach used in BioFabric, the edges incident on a node are, by design, distributed along the *full length* of the horizontal node line. One disadvantage of this approach is that an edge is more closely tied visually with only one of the endpoint nodes, and can be conceptually disconnected from the other node. But as shown in Figure 2, BioFabric can address this by creating *shadow links*; every edge in the network is simply duplicated, with the prefix “shdw” added to the relation label for the link. Then, one of this pair of links is associated with each node, with the “real” link always showing up to the left of the shadow link, more closely associated with the node in the upper row. This presentation allows the user to see the full inventory of the edges incident on a node in a single compact presentation, and the full set of edges for two or more nodes can be directly compared visually. This mode is chosen from the ***Set Display Options*** dialog box. The disadvantages of this approach are that the width of the network doubles, and the distinct shape of the network outline, which is often a useful tool for navigation and user intuition, is obscured.

### Link grouping

*Link grouping* is a BioFabric feature that leverages both the wide flexibility for assigning columns to network edges, as well as the advantage of edge wedges for highlighting differences in node connectivity. If the user has assigning unique suffix tags to the link relation descriptors that partition the edges into distinct sets, BioFabric can use these tags to order and layout the edges incident on each node according to this scheme. As Case Study III will illustrate below, this allows the user to unambiguously and directly compare how the connectivity of a node, or a set of nodes, varies across multiple networks.

### Layout algorithms

A network layout for BioFabric is very simple, and just consists of: 1) the linear ordering of the *n* nodes, assigned to rows 1 to *n*, and 2) the linear ordering of the *e* edges, assigned to columns 1 to *e*. But this simple framework still provides a variety of different, powerful ways to organize a complex network.

### Default layout

The default layout was designed to provide a fast technique for organizing the network in an understandable and useful fashion. It is simply a breadth-first traversal of the network from most connected component, where the neighboring nodes are visited in the order determined by their degree. The network shown previously in Figure 1 has been laid out using this technique. Some general principles are:

•The algorithm works in two passes, where the node rows are assigned first, followed by the edge columns.

•All edges are treated as undirected, even with directed networks.

•Duplicate edges (i.e. with identical endpoints but different relation labels) are ignored when calculating node degree.

•Ties are broken using lexicographic ordering of node names.

For the base case (no shadow links or link groups) the algorithm proceeds as follows:

Node assignment:

1. Set row 1 as the next available row.

2. Find the highest degree node not yet processed, and assign it to the next available row. Make that row the current row; increment the next available row.

3. Take the node assigned to the current row and order its neighbors based upon their degree, highest degree first.

4. Traversing the neighbor nodes using that order, if the node has not yet been assigned, assign it to the next available row and increment the next available row.

5. Increment the current row. If a node has been assigned to that row, go to step 3. If not, go to step 2.

Edge assignment:

1. Set column 1 as the next available column. Make row 1 the current row *c*.

2. For current row *c*, get all the unassigned edges for the node in that row. Note that since we are not dealing with shadow links, all unassigned edges must connect to rows ≥ *c*.

3. For each row *r* ≥ *c*, create a set *S* of edges incident on *c* and *r*. Order these sets by increasing row number *r*, so that edges will be assigned in order of increasing length.

4. Iterating through the ordered list of sets, for each set *S*, order those edges in *S* based on lexicographic ordering of the link relation description, and assign them to the next available columns in this order; increment next available column appropriately. If there is a pair of directed edges with the same link relation description, downward links are assigned before upward links.

5. Increment the current row, and go to step 2.

### Connectivity layout

**Figure 3:**
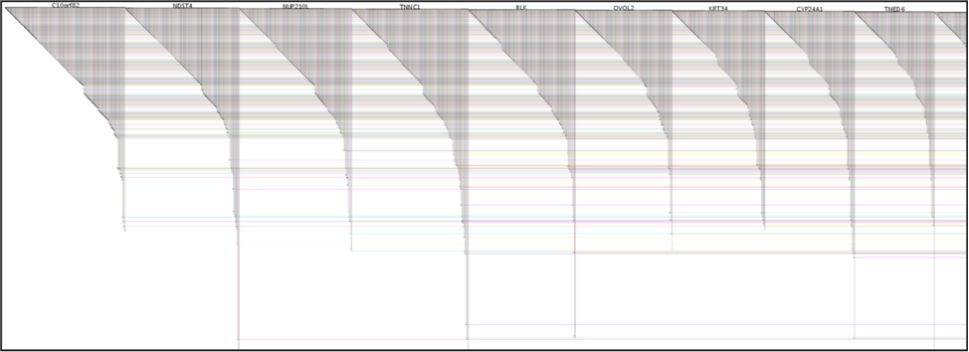
**Connectivity layout.** This layout technique tries to place nodes with similar connectivity next to each other in the linear ordering of nodes. The results are extended runs of nodes that appear similar, i.e. have roughly the same *edge wedges*. The layout is less compact than the default; the long tails exiting the bottom of the figure have been cut off to conserve space.

One of the characteristics of a BioFabric network is that it creates a linear ordering of the nodes, and sometimes it can be useful to be able to visually compare sets of nodes with similar connectivity, thereby being able to quickly assess the similarities and differences between these nodes. BioFabric’s connectivity layout is designed to support this capability; Figure 3 shows a detail of a network laid out in this fashion.

For a network of *n* nodes and *e* edges, the algorithm first tags each edge with a coefficient that represents the similarity between the connectivities of the two endpoints nodes. Two methods are available: cosine similarity [12] or Jaccard similarity [13]. Note that in both cases, directed edges are treated as undirected, so the similarity coefficients are symmetric.

#### Cosine similarity

Each node *m* has associated connectivity *n*-vector *C*_*m*_ = (*x*_1_, *x*_2_, … *x*_*n*_) where *x*_*j*_ = 1.0 iff the network has an edge (*j*, *m*) or (*m*, *j*), else *x*_*j*_ = 0.0. For an edge *e*: (*j*, *m*), the cosine similarity *S*_*e*_ is:

(1) $$S_{e}=\left(C_{j}\cdot C_{m}\right)/\left(\left\|C_{j}\right\|,\left\|C_{m}\right\|\right)$$

#### Jaccard similarity

Each node *m* of degree *d* has an associated connectivity set of neighbor nodes *N*_*m*_ = (*n*_1_, *n*_2_, … *n*_*d*_). For an edge *e*: (*j*, *m*), the *Jaccard* similarity *S*_*e*_ is:

(2) $$S_{e}=|N_{j}\cap N_{m}\left|/\right|N_{j}\cup N_{m}|$$

Nodes are brought into the set of placed nodes *P* one at a time, only considering nodes from the front *F*, which is the subset of nodes in the set of unplaced nodes *U* that have at least one edge to a node in *P*. A simple approach would be to select a node from *F* with the highest similarity coefficient of all the edges from *P* to *F*. But if the algorithm is in the process of “mining” a region of the network that is richly interconnected, the simple approach would tend to easily abandon this growing chain of similarly connected nodes if a slightly higher similarity coefficient appears anywhere else along the front. To create longer runs of similar nodes, it is preferable to make the algorithm “sticky”.

To achieve this, the algorithm maintains an ordered *chain* of the *r* most recently used nodes, as well as a threshold fraction 0.0 ≤ *t* ≤ 1.0; both these values *r* and *t* are user-configurable. If the highest coefficient *S*_*b*_ to the front is assigned to a link from node *A*, but there is a coefficient *S*_*d*_ assigned to an edge from node *C* in the chain to the front, such that *S*_*d*_ >*S*_*b*_ * *t*, the node connected to *C* would be added to the placed set *P*. Otherwise, if the node in the front connected to *A* wins and is placed in *P*, the algorithm empties the current chain. Regardless, the connected node (*A* or *C*) in *P* is either added in the first slot, or (if *C*) moved up to the first slot of the chain, and the newly added node is inserted into the second slot in the chain, pushing all other elements back. If the new addition causes the chain to exceed the maximum size, the least recently accessed node is removed from end of the chain.

### Interoperation with other software tools

Cytoscape [2] is a powerful and popular platform for analyzing networks, and the platform supports an extensive ecosystem of users and plug-in developers, so it is highly desirable to be able to leverage this platform. The Gaggle [14] is a software system that allows users to exchange data between heterogeneous, independent software tools, and the CyGoose plug-in allows Cytoscape to work with Gaggle. Since BioFabric is a tool that supports a unique way of visualizing, navigating, and exploring networks, but is not a tool for supporting computational analysis, it has been Gaggle-enabled to allow it to work with, and leverage the strengths of, these other analysis tools. Using Gaggle, networks and selections can be exchanged between BioFabric and other Gaggle-aware tools running on the user’s desktop. To support this, a Gaggle-aware version of BioFabric can be launched from the BioFabric web site using Java Web Start.

## Results and discussion

### BioFabric advantages

The following four case studies highlight the advantages of using BioFabric to explore large networks. Some of these advantages are:

•The ability to use a single, coherent, rational, unambiguous layout of an entire large network as a basis for navigating and exploring that network.

•A means of quickly assessing the connectivity of nodes through the depicted *edge wedges*.

•A superior way of unambiguously depicting the edge relationships in clustered networks.

•A way of visually identifying differences in network connectivity between multiple conditions through the use of link grouping and the connectivity layout.

•The ability to identify interesting network structures and properties at large scales through simple inspection.

### Networks need to first be imported into BioFabric

The current incarnation of BioFabric is designed to be a network viewer, not an editor, and thus networks need to be first imported either as a Cytoscape *tab*-*delimited*. sif file, or using the Gaggle network import method described above. In order to retain the final chosen layout and display options, the network can then be saved and reloaded as a BioFabric .bif file, which is an XML-based format.

### Case study I: Introduction to the BioFabric interface using a large network

**Figure 4:**
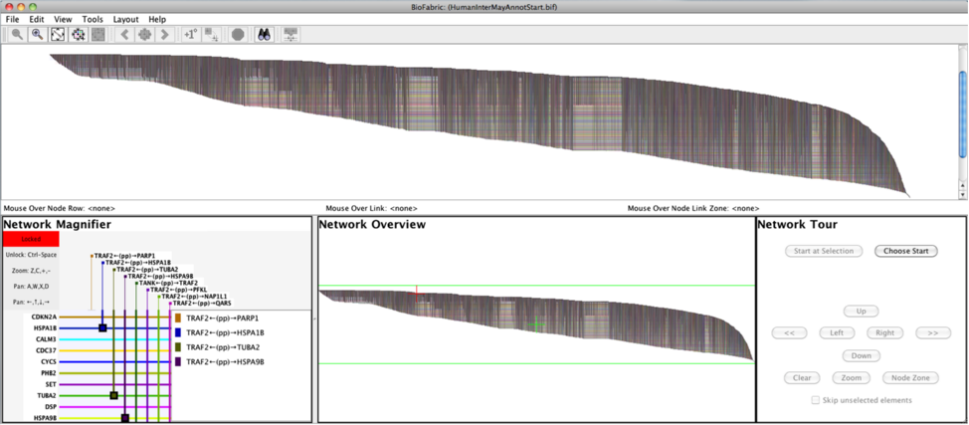
**Screenshot of BioFabric.** This shows the Human_Interactome_May.sif [15] network after it has been loaded and arranged using the default layout algorithm. This is a network of over 10,000 nodes and 61,000 links. BioFabric augments the full-network view (top pane) with a mouse location bar (thin strip directly below the full-network view), a network magnifier (lower left), a fixed overview (lower center), and a touring tool (lower right). The magnifier can either automatically track the mouse position or be locked down; it provides detailed information about areas of interest. The overview provides guidance on the location of the current view of the network, as well as current mouse position and current magnifier view. The touring tool allows the user to systematically traverse network links and step along node rows to explore the network.

To illustrate how BioFabric can be used to explore a large-size network, we will use the sample network data file Human_Interactome_May.sif [15] available from the Cytoscape web site; this file is described as combining interactions reported in several databases [16-20] and papers [21-23]. The network has over 10,000 nodes and 61,000 links. By using the ***File*** → ***Import*** → ***Import SIF with Node Attributes***… command, the network definition can be imported simultaneously with the accompanying annotation file that supplies node names (which was first edited to remove rows with missing first-column entries). Once it is loaded, and the directionality of edge relationships is specified in a pop-up dialog, the BioFabric application appears as in Figure 4. Note that BioFabric does not display duplicate edges, and so one of the edges in the .sif file gets dropped during the load.

In addition to the main network presentation panel, BioFabric contains four other features in the main window:

1. **Mouse Location**: This thin bar is located immediately under the main network view, and reports the node row, link column, and node link zone currently under the mouse. In most cases, the node link zone can be thought of as the node associated with the *edge wedge* currently under the mouse.

2. **Network Magnifier**: This gives a magnified view of the network under the mouse, along with a listing of all the links that display an *endpoint glyph* in that magnified view. The magnification can be easily varied; at maximum magnification, detailed information about the visible link ends and nodes are shown on the view boundary. The magnifier is manipulated using the displayed key shortcuts, so it can be operated simultaneously alongside the mouse. When desired, the magnifier can be locked, thereby disconnecting it from the mouse, and panned and zoomed independently.

3. **Network Overview**: This panel always shows a fixed full-network view, while the current viewport, mouse location, and (possibly locked) magnifier location are shown in context.

4. **Network Tour**: This panel drives the network tour feature. The user can select a link endpoint, and then navigate orthogonally through the network. For example, buttons allow the user to jump along the current node row between adjacent link endpoints, or from one end of a link to the other. This tool allows the network features to be explored in a systematic, organized fashion.

Note that Figure 4 demonstrates that even zoomed out to the full network level, some features of the network stand out. For example, there are long, clearly visible stretches of similarly interacting proteins that turn out to be, for example, ribosomal proteins or RNA polymerase proteins.

**Figure 5:**
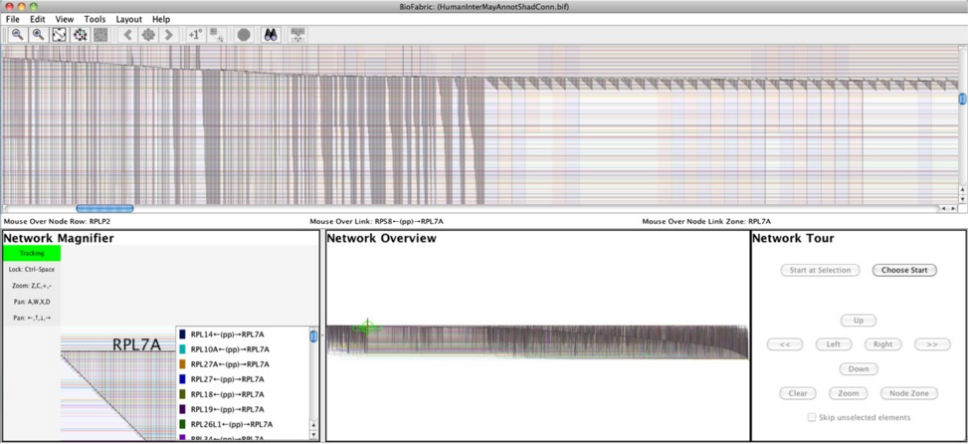
**Network with shadow links**, **connectivity layout**, **and node zone shading.** The interactome network has now been reorganized using shadow links and the connectivity layout. Also note that node zone shading has been activated, providing contrasting light backgrounds behind each node zone. Contrast the network overview here with that in Figure 5; the overall network organization is less compact, but the small-scale organization of nodes allows direct comparison of similar proteins. The network magnifier has been set to a medium zoom level to browse the current viewport; note how the overview shows our current location in the full network.

Figure 5 shows the end result of a series of logical next steps after importing a network, which is to turn on shadow links and apply the connectivity layout; Additional file 1 is the BioFabric file for the resulting network. Another useful feature that is shown in this view is *node zone shading*, which applies alternating light blue and light pink backgrounds to the node zones. This makes the edges associated with each node stand out even at large scales. It is informative to compare the network overview displays between Figure 4 and Figure 5, thereby showing how shadow links and the connectivity layout change the overall network appearance. The clean shape and compactness of the default, standard presentation is gone, but the relationship between related nodes is clearer.

**Figure 6:**
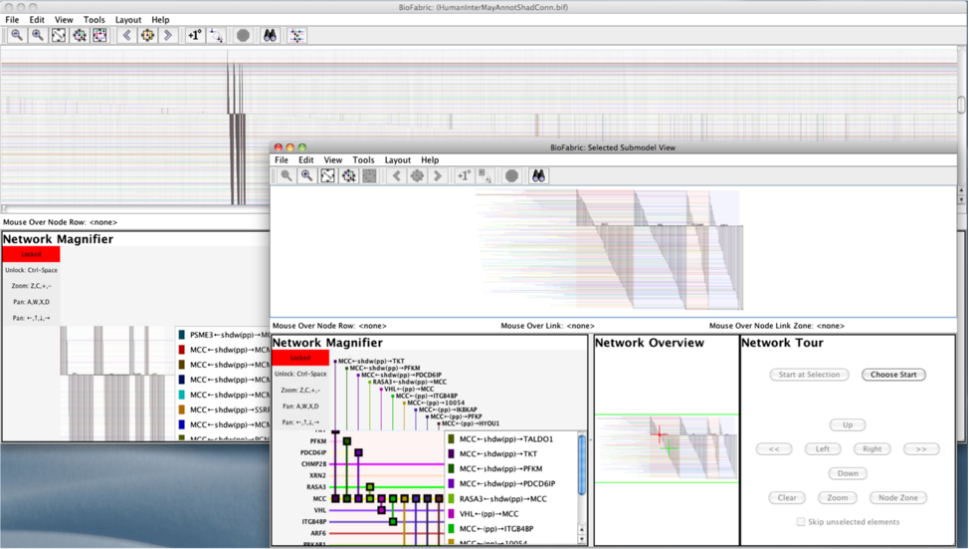
**Creating a subset view.** Four adjacent proteins in the interactome network were selected. Clicking on the first-neighbor button in the toolbar then selected the neighbor nodes and connecting edges; this selection pattern is visible in the main window in the background. Clicking on the ***Send Selections to Subset View*** button on the right of the toolbar creates the view shown in the foreground. This compact view omits all unused rows and columns while otherwise retaining the layout used in the main network; the option to omit duplicate shadow and true links has also been enabled. Note that the magnifier in the foreground subset view has been set to maximum zoom, so that edges and nodes are fully labeled in the magnifier.

A subset view is then shown in Figure 6. This is a very useful tool for directly comparing nodes that may be widely separated in the main layout. Even with runs of adjacent nodes, this view provides the valuable service of displaying a compact representation that squeezes out all the irrelevant rows and columns, while still retaining the exact relative positioning of all the network elements. To launch this subset view, the user does the following:

1. Find interesting nodes, either by browsing or using the search tool. Select each node either by clicking on the node row, or the node name. If using search, the results are selected already.

2. Click on the ***Add First Neighbors to Selection*** button on the toolbar, which adds the neighboring nodes, as well as the connecting edges, to the current selection.

3. Click on the ***Send Selections to Subset View*** button on the toolbar.

4. The subset view appears in a separate window, which behaves just like the main window, except that only one level of subset view creation is currently supported.

**Figure 7:**
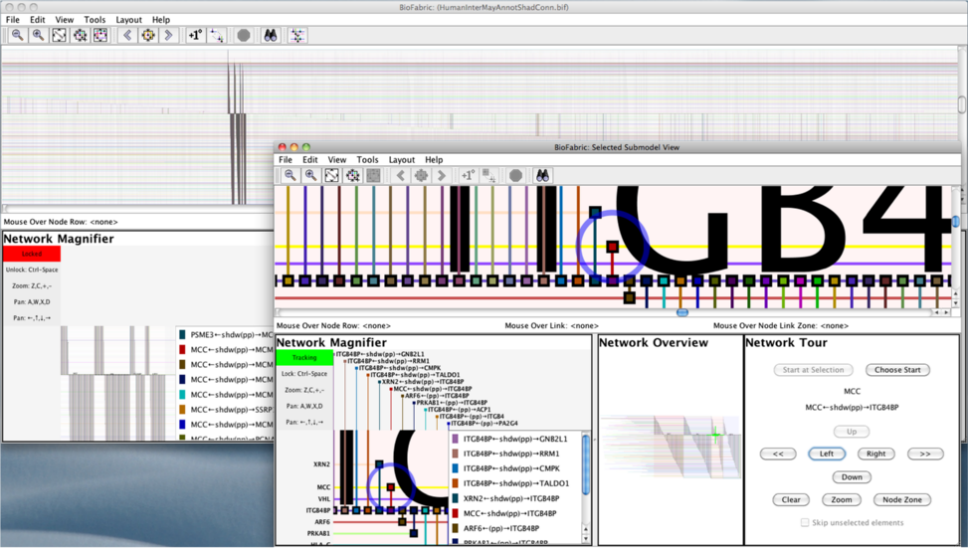
**Network tour of the subset view.** The user can start a network tour by clicking on the ***Choose Start*** button and then clicking on an edge endpoint (colored square glyph). Then, the buttons in the Network Tour panel can be used to move left or right along the current node row, or up and down along the current edge column. The tour panel displays the current node and edge; note the selected edge is a shadow link, as indicated by the prefix on the link relation tag.

Finally, Figure 7 shows a network tour in progress. The user starts a tour either by clicking the ***Choose Start*** button and clicking on an edge endpoint, or (if a node is already selected) the ***Start at Selection*** button in the Network Tour panel. The current tour location is then indicated by a blue circle, and is also textually described in the tour panel. The tour panel buttons allow the user to either navigate to other edges incident on the current node (by moving left or right), or to navigate to the distal node for the current edge (by either moving up or down). The user can change the zoom level as needed during the tour to maintain the desired level of context, but can always return to the exact current tour location using the tour ***Zoom*** button.

### Case study II: Understanding clustered networks

The traditional network layout method is frequently used to depict the results of applying network clustering algorithms. While the proximity of clustered nodes provides a useful visualization, the edges are typically cluttered, so that the user cannot see the internal edge structure of the cluster, nor can she see where inter-cluster edges terminate. Furthermore, edges just passing through a cluster can be mistaken as representing a non-existent relationship between clusters.

**Figure 8:**
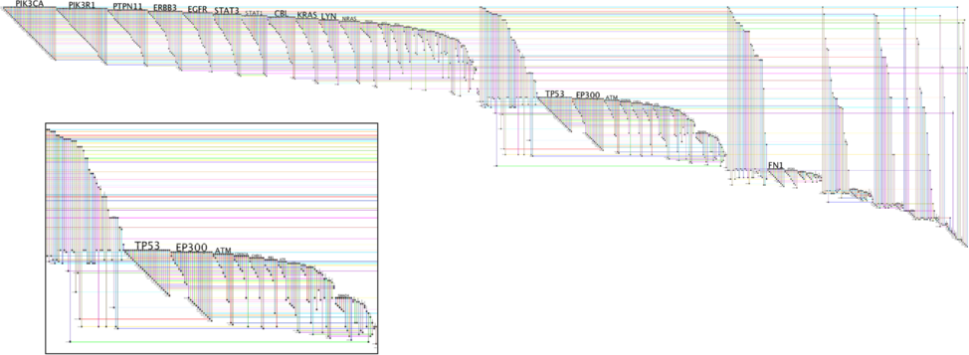
**Clustered network shown in BioFabric.** This is a BioFabric version of the network presented in Figure 4 of [24]. The main image shows the whole network, while the inset shows a magnified version of Cluster 1. Note how the ability to group edges horizontally allows inter- and intra-cluster edges to be segregated for clarity. As the inset shows, the nodes in Cluster 1 that are the targets of the edges coming from Cluster 0 can be easily discerned.

BioFabric’s ability to segregate links into bundles of distinct functionality along the horizontal axis can instead create a clear and unambiguous representation of a clustered network. To illustrate this, we will use a network depicted in Figure 4 of [24], which presents clustering results for altered genes from The Cancer Genome Atlas (TCGA) data set applied to their underlying functional protein interaction network. A BioFabric version of this network is shown in Figure 8. To create this presentation, the required node and link orderings were generated and then specified in two files, which were imported using the ***Layout*** → ***Layout Using Node Attributes*** command followed by the ***Layout*** → ***Layout Using Link Attributes*** command. This is necessary because BioFabric does not yet have a built-in cluster layout algorithm. However, this layout was prepared externally by applying the default layout to each cluster separately, ordering the clusters by the cluster number used in the original analysis [24], and assigning the remaining inter-cluster edges to the appropriate interstices between each cluster. Two crucial aspects of using BioFabric for presenting clustered networks stand out:

•Nodes and internal edges in a cluster can be assigned to contiguous sets of rows and columns, creating clear and concise depictions of each cluster as it stands as an independent sub-network.

•The edges connecting clusters are shown as discrete bundles, completely separated from intra-cluster edges, and are assigned to target clusters in a logical, ordered fashion. Edge endpoints are not obscured, allowing any primary inter-cluster hubs in each cluster to appear clearly in the depiction. Additionally, there are no ambiguous inter-cluster edges that can create the false impression that two clusters are directly linked.

### Case study III: Visualizing the differences between cancer subtypes

The Center for Systems Analysis of the Cancer Regulome (CSACR) website [25] provides a wealth of TCGA cancer data, such as analyses of significant pairwise feature associations iidentified via standard statistical tests. These features are heterogeneous, and can include quantities such as gene expression, mutations, copy number variations, and clinical outcomes. By constructing networks of these associations, researchers can study how these heterogeneous features interact in the various cancer types.

One type of cancer studied is glioblastoma multiforme (GBM) [26], of which there are four different subtypes: classical, mesenchymal, neural, and proneural [27]. Separate CSACR pairwise feature association studies have been carried out for these four types, as well as a unified study that combines all four [28-32]. This case study will use these data to demonstrate how BioFabric can graphically compare the differences between a set of networks; i.e. the differences in associations between these GBM subtypes. This example also illustrates how the researcher can visualize and linearly browse a very large network. Of course, the best way to actually find a comprehensive list of these differences at this scale is not to browse this network, but to use computational tools that calculate and compare node degree across the subtypes.

**Figure 9:**
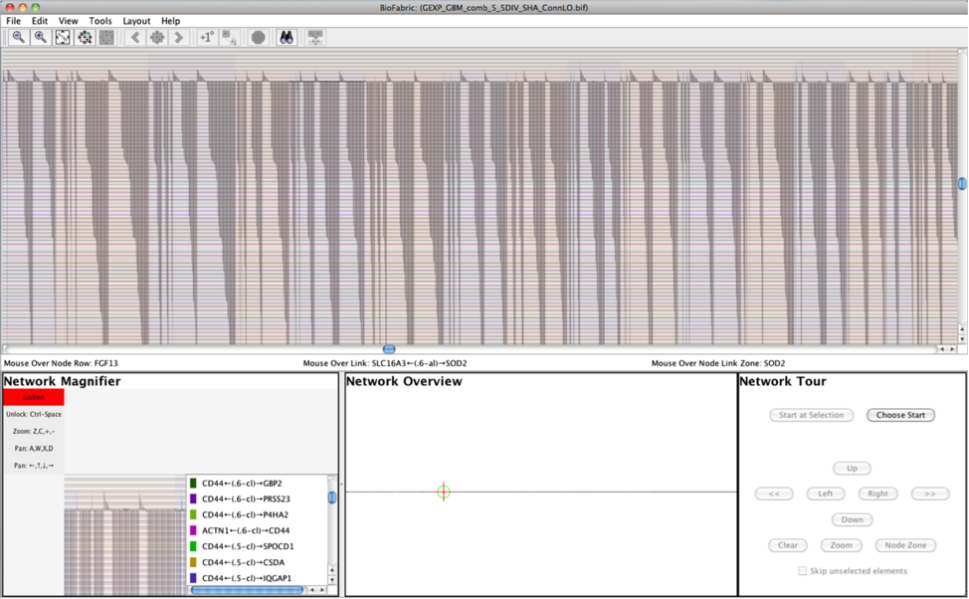
**Browsing TCGA paired associations network with grouped links.** This network of paired associations for four different GBM subtypes (plus a fifth combined analysis) contains over 5,000 nodes and 10^6^ edges. The *link grouping* feature is being used, so that each node typically shows five ordered, distinct *edge wedges*, one for each of the analyses. The alternating light blue/pink *node zone shading* feature allows these wedges groups to be associated with the underlying node. In this case, dragging the mouse while holding down the *Ctrl* (*Command* on Mac) key, in combination with the network magnifier, provides a simple way to browse the entire network in a linear fashion, looking for interesting patterns between the subtypes.

This example uses the pairwise associations of gene expression levels from the five different analyses, taking just those associations with a correlation coefficient of absolute value ≥ 0.5. These were all combined into a single network comprised of over 5,000 nodes and 10^6^ edges, which were written to a .sif file and imported into BioFabric. Additional file 2 is a BioFabric file for a reduced-size version of the resulting network, with a coefficient threshold of 0.6. The full-size network file used here is available from the BioFabric web site. Shadow links were activated (thereby generating a network display of over 2x10^6^ links), and the connectivity layout was applied. Most importantly, the edges for each of the five different studies were annotated with a unique tag, and this tag was used to group the edge using the previously described BioFabric link grouping feature; the edge wedges of the five analyses are ordered left-to-right in the order listed above. The result is shown in Figure 9. As the BioFabric network overview panel in the figure implies, little can be surmised from this particular full-network view, which has an aspect ratio of 0.0025. However, this vast network is now represented as basically a linear, sequential catalogue. The connectivity layout has usefully imposed a systematic low-level structure onto this very large network, as nodes with similar connectivity are located adjacent to one another. Furthermore, the node zone shading feature, in combination with link grouping, helps the user browse the different association patterns for each gene, as each gene typically shows five separate wedges, one for each analysis. Figure 9 demonstrates how it is possible to simultaneously visualize the different association patterns across the subtypes for a large number of nodes even at the global scale. The user can slide the scrollbar, or drag the mouse while holding down the *Ctrl* (*Command* for Mac) key, and zoom in with the network magnifier, to systematically browse any part of the entire network in a linear fashion.

**Figure 10:**
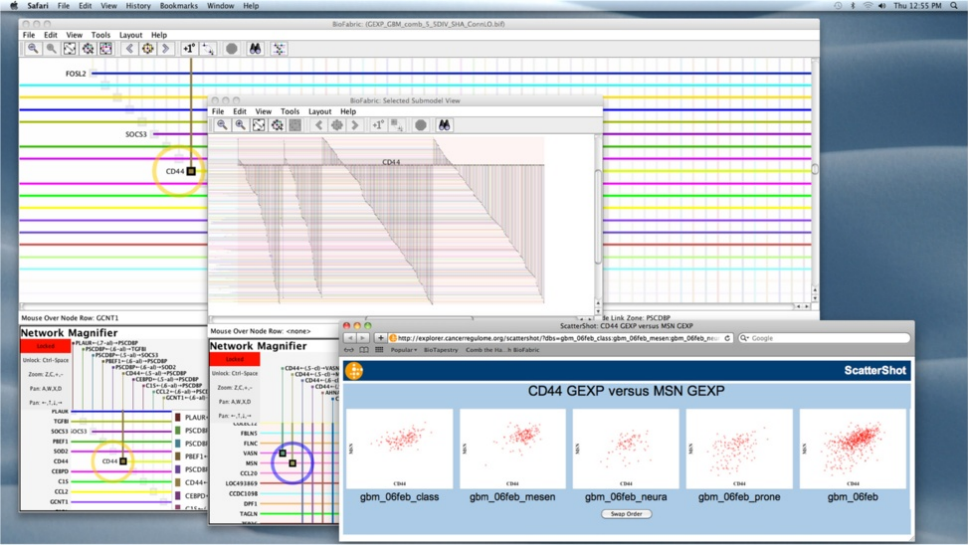
**BioFabric working in concert with a web application.** Simple visual inspection of the *CD44* gene shows that there are almost no pairwise associations at or above the 0.5 cutoff threshold for the GBM mesenchymal subtype, as only four edge wedges are prominent. By generating a subset view for *CD44* (middle window above) and its first neighbors, the presence or absence of an association for the various subtypes can be quickly scanned by eye. By right-clicking on an edge endpoint, the user launches a web application in the web browser that simultaneously shows the scatterplots for the five different analyses (lower right).

To show how networks can be visually compared in detail, we will focus on *CD44*, which is known to be overexpressed in the mesenchymal subtype [33]. Creating a first-neighbor subset network for *CD44*, as shown in Figure 10, indeed reveals that only three of the subtypes, plus the unified analysis, seem to appear. On close inspection, the mesenchymal subtype does appear, but it consists of a thin wedge of only six associations. This is not necessarily unexpected for this analysis, since detecting a pairwise association of *CD44* expression with other genes requires appreciable variation among the different patient samples. *CD44* expression consistently fixed near a high level might therefore be expected to lead to a reduction of the number of pairwise associations.

Exploring this *CD44* subset model, the edge wedge shapes help to spot differences between the subtypes, and the presence or absence of an association for each of the various subtypes can be quickly scanned left-to-right along any node line. For any association, right-clicking on a *link endpoint* allows the user to launch a web browser for a user-defined hyperlink that has been previously specified in the ***Edit Display Options*** dialog. (Note that this is in contrast to right-clicking on a node line, which launches a web browser for the associated node.)

In this particular example, a right-click launches a web application built on top of the CSACR Regulome Explorer data portal [34] that queries the TCGA database and displays scatterplots of the underlying data for the five different analyses. This particular association shown in the figure, between the gene expression levels of *CD44* and *MSN*, actually only appears in the network for the classical and unified analyses; inspecting and comparing the different scatterplots provides insights into why this is the case.

### Case study IV: Full-network shapes with the default layout

**Figure 11:**
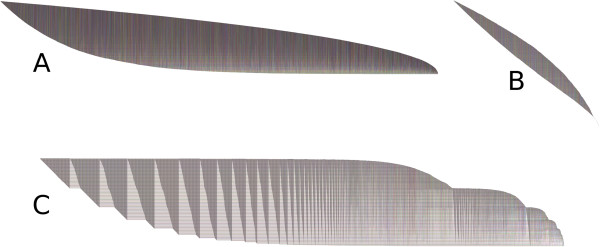
**Largescale network properties revealed by shape.A**) Undirected Erdos-Renyi graph with 10K nodes and 60K edges; **B**) undirected Erdos-Renyi graph with 10K nodes and 10K edges; **C**) undirected Barabasi-Albert scale-free graph with 2K nodes, six edges added each time step, generated using psumtree algorithm. All were generated using the R igraph v0.6 package [35] and laid out using BioFabric default layout technique. These networks are not shown to the same scale. The shapes of the lower and upper boundaries, and large-scale edge patterns show basic network properties; see text for discussion.

Recall that the BioFabric default layout algorithm is simply a breadth-first traversal of the network from the most connected component, where the neighboring nodes are visited in the order determined by their degree. Keeping this in mind, a quick glance at a network that is laid out using this algorithm can provide useful insights into the structure and global properties of the network. To illustrate this, Figure 11 presents three different random networks, which were all generated using the R igraph v0.6 package [35]. The BioFabric files for these three networks are included in Additional file 3. The first two networks are undirected Erdos-Renyi random graphs [36] with 10^4^ nodes; network A has 60,000 edges, while network B has only 10^4^ edges. In contrast, network C is an undirected Barabasi-Albert scale-free graph [37] with 2,000 nodes and almost 12,000 edges. As would be expected, networks A and B show no discernable edge patterns, while the scale-free network shows a distinct sawtooth pattern for the edge wedges. A quick perusal of the network C edge wedges also allows the viewer to quickly estimate the fraction of previously visited and unvisited nodes being encountered at each step of the breadth-first search used in the layout.

BioFabric lays out node rows and edge columns using a fixed, square grid. This feature means that the *slopes* of the upper and lower boundaries also provide visual clues about network structure. In particular, when the lower boundary is at a 45-degree angle, each newly added edge is adding one new node. But where the slope is zero degrees, each newly added edge is incident on a previous visited node. Thus, network B, which has the same number of nodes as edges, has a lower boundary slope that is unsurprisingly approaching the 45-degree limit.

### Current limitations of BioFabric

BioFabric’s pervasive use of its fundamental underlying abstraction of nodes and edges as simple orthogonal lines has a significant advantage in being able to consistently represent a network at all scales. However, this approach does result in a very simple, abstract representation of the network, and so it currently lacks the expressive power that is available through the traditional method of representing networks when used on networks of medium size or smaller. For example, one area where these limitations are apparent is the representation of signalling and metabolic pathways, where rich symbol libraries for nodes and edges can succinctly convey significant amounts of information. The flexibility afforded by the traditional technique also means that important features such as information flow and paths (including parallel paths and cycles) can be given particular emphasis for clarity, so such features can be more difficult to identify in a BioFabric presentation.

Perhaps some or all of these limitations can be addressed through further extensions to BioFabric, including the additional development of new layouts techniques and tools for interactively investigating and illustrating network structures such as paths. These limitations can also be sidestepped if BioFabric’s presentation technique were more tightly integrated as a complement to traditional techniques. Allowing the researcher to toggle between traditional and BioFabric visualizations inside a single tool such as Cytoscape could do this, for example.

### Future work

Much work remains to be done to leverage the new visualization technique introduced by BioFabric, including improvements to the usability, scalability, and feature set of the first-generation implementation. Some particular directions to pursue include:

•Introducing compact representations of network motifs such as cliques.

•Investigating new layout algorithms, perhaps applying existing heuristic algorithms for the linear arrangement problem, bandwidth reduction, and profile reduction [6].

•Extending the representation of nodes as lines in two dimensions into representing them as planes in three dimensions.

•Incorporating a model hierarchy into the software, in a manner similar to that used in BioTapestry [9,10]. This will allow complex models to be systematically organized into relevant subsystems.

•Implementing navigational features, such as bookmarks, that leverage BioFabric’s presentation of a network as an extended sequential representation.

•Implementing metanodes to allow BioFabric to support more complex network models.

•Providing additional layouts methods and interactive tools to help the researcher better visualize network features such as paths (including parallel paths and cycles). Improving the network magnifier to give a more visual (as opposed to textual) sense of first neighbors will also help to provide a more intuitive sense of connectivity.

•Porting the technique into browser-based technologies such as HTML5 Canvas may prove challenging given the demanding graphics requirements, but will allow the method to be used by the emerging class of purely browser-based web applications.

Finally, since the advantages of BioFabric can be complementary to the advantages provided by traditional network presentation techniques, a combination of the two should provide the most expressive power. The new Cytoscape version 3.0 is designed to support alternate renderers (e.g. [38]), and this provides an avenue for creating such a combined tool. It would also be fruitful to investigate how one could seamlessly move back and forth between the two types of representations.

## Conclusions

BioFabric is a new network visualization software application that challenges the traditional underlying concept of how network nodes and edges are represented in two-dimensional space. In doing so, it gives researchers a powerful tool that provides an organized, comprehensible, scalable way of visualizing large and complex networks.

## Availability and requirements

**Project Name**: BioFabric

**Project Home Page**: http://www.BioFabric.org/index.html

**Operating Systems**: Cross-platform. Windows and Mac Version 1.0.0 executables are provided in Additional files 4 and 5, respectively. Download the most current executables from the project home page.

**Programming Language**: Java

**Other Requirements**: Minimum requirement is Java 5, although code outside of the Gaggle subsystem can be compiled using Java 1.4 if desired. The large network presented in Case Study III required the Java heap allocation to be set to 4 gigabytes to import and layout, with the corresponding appropriate hardware. On Mac OS X, Java 6 is required to render the networks with the desired brightness.

**License**: LGPL V 2.1. Some of the toolbar image files are freely distributed under a separate license from Sun Microsystems, now Oracle. The launch4j wrapper [39] used to create the Windows executable is licensed under the BSD and MIT licenses. The author of the code forming the basis for browser launching [40] has declared it to be public domain. Per the LGPL license, the source code for Version 1.0.0 is provided in Additional file 6.

**Any restrictions to use by non**-**academics**: None

## Competing interests

The author declares that he has no competing interests.

## Authors’ contributions

WJRL conceived, designed, and wrote BioFabric, developed the case studies, and wrote the manuscript.

## Supplementary Material

Additional file 1 **File Format: ZIP archive containing a BioFabric.bif (XML format) file.** Title of Data: Human Interactome Network for Case Study I. Description of Data: This is the BioFabric file of the network built from the Human_Interactome_May.sif file and associated node annotations file obtained from [15] and shown in Case Study I. Unzip the file and extract the HumanInteractomeMayAnnotShadConn.bif file (19 MB), which can be loaded into BioFabric. Click here for file

Additional file 2 **File Format: ZIP archive containing a BioFabric .bif (XML format) file.** Title of Data: Reduced Network for Case Study III. Description of Data: This is a *reduced* version of the BioFabric file containing the TCGA CSACR network of paired gene expression associations for four different GBM subtypes (plus a fifth unified analysis) used in Case Study III. The full file could not be included due to space limitations, so this only contains correlations with an absolute value ≥ 0.6 (instead of 0.5 used in the example). However, as the illustrated *CD44*-*MSN* associations have a correlation coefficient of 0.5, they are not present in this file. Unzip the file and extract the GEXP_GBM_comb_6_5DIV_SHA_ConnLO.bif file (82 MB), which can then be loaded into BioFabric. The full file can be downloaded from the BioFabric project web site. Click here for file

Additional file 3 **File Format: ZIP archive containing three BioFabric.bif (XML format) files.** Title of Data: Random Networks for Case Study IV. Description of Data: These are the three random networks shown in Case Study IV. Unzip the file and extract the three files (er1060.bif, er1010.bif, ba2K.bif), each can then be loaded into BioFabric. Click here for file

Additional file 4 **File Format: ZIP archive containing the version 1.0.0 BioFabric.exe executable for Windows computers.** Title of Data: BioFabric Windows Executable. Description of Data: This contains the BioFabric application bundled for Windows, configured with a maximum Java heap space of 1 GB. As this is version 1.0.0, it is preferable to download the latest version of BioFabric from the project web site. Click here for file

Additional file 5 **File Format: Mac Disk Image.** Title of Data: BioFabric Mac OS X Executable. Description of Data: This disk image contains the BioFabric application bundled for Mac OS X, configured with a maximum Java heap space of 1 GB. As this is version 1.0.0, it is preferable to download the latest version of BioFabric from the project web site. Click here for file

Additional file 6 **File Format: Gzipped tar file containing packages of Java source code, image, and property files.** Title of Data: Version 1.0.0 BioFabric Source Code. Description of Data: This file contains the source code needed to build BioFabric. If Gaggle support is not needed, it can be compiled with Java 1.4. Click here for file
